# Evidence against a blood derived origin for transforming growth factor beta induced protein in corneal disorders caused by mutations in the *TGFBI* gene

**Published:** 2007-06-27

**Authors:** Henrik Karring, Zuzana Valnickova, Ida B. Thøgersen, Chris J. Hedegaard, Torben Møller-Pedersen, Torsten Kristensen, Gordon K. Klintworth, Jan J. Enghild

**Affiliations:** 1 Center for Insoluble Protein Structures (inSPIN) at the Department of Molecular Biology, Science Park, University of Aarhus, Aarhus C, Denmark; 2 Institute for Inflammation Research (IIR), 7521, National University Hospital (Rigshospitalet), Copenhagen Ø, Denmark; 3 Departments of Pathology and Ophthalmology, Duke University Medical Center, Durham, NC

## Abstract

**Purpose:**

Several inherited corneal disorders in humans result from mutations in the transforming growth factor beta induced gene (*TGFBI*), which encodes for the extracellular transforming growth factor beta induced protein (TGFBIp) that is one of the most abundant proteins in the cornea. We previously reported a significant amount of TGFBIp in plasma by immunoblotting using the only TGFBIp antiserum (anti-p68^βig-h3^) available at that time (anti-p68^βig-h3^ was generated against residues Val_210_-His_683_ of TGFBIp). This observation raised the possibility that a fraction of corneal TGFBIp may originate from the plasma. However, recent experiments in our laboratory indicated that the anti-p68^βig-h3^ antiserum cross-reacts with an environmental protein contaminant. Therefore, we investigated the specificity of the originally utilized anti-p68^βig-h3^ antiserum and re-evaluated the amount of TGFBIp in human plasma by immunoblotting using a new specific antiserum.

**Methods:**

The observed cross-reactivity of the previously utilized anti-p68^βig-h3^ antiserum was tested by immunoblotting and the antigen identity was determined by mass spectrometry. A part of human *TGFBI* encoding an NH_2_-terminal 11.4 kDa fragment of TGFBIp (residues Gly_134_-Ile_236_) was amplified by polymerase chain reaction (PCR) and cloned in *E. coli*. The TGFBIp fragment was expressed in *E. coli*, purified by Ni^2+^-affinity chromatography, and used to immunize rabbits to produce a specific antiserum (anti-TGFBIp^134-236^). To enhance the detection of possible TGFBIp in plasma by allowing a higher sample load, albumin and immunoglobulin G (IgG) were specifically depleted from normal human plasma by affinity chromatography. The presence of TGFBIp in plasma was investigated by immunoblotting using the anti-TGFBIp^134-236^ antiserum. Purified TGFBIp from porcine corneas was used for estimation of the TGFBIp detection limit.

**Results:**

The previously utilized TGFBIp antiserum, anti-p68^βig-h3^, cross-reacted with human keratin-1, a common environmental protein contaminant. Thus, the anti-p68^βig-h3^ antiserum recognizes both TGFBIp and keratin-1. In contrast, the anti-TGFBIp^134-236^ antiserum reacted with TGFBIp but showed no indication of reactivity with other proteins in plasma. Using this antiserum, TGFBIp was not detected in crude or albumin/IgG-depleted human plasma and the detection limit of TGFBIp using immunoblotting was estimated to be 10 ng.

**Conclusions:**

Our failure to detect TGFBIp in human plasma using a highly specific antiserum suggests that TGFBIp is not present in a physiologically relevant concentration in human plasma. The previous impression that normal human plasma contains a significant amount of TGFBIp by immunoblotting was due to the utilization of a less specific antiserum that recognizes both TGFBIp and human keratin-1. Together with other results, our observation makes it unlikely that TGFBIp is imported into the cornea from the circulation as reported for other abundant extracellular corneal proteins and suggests corneal origin of TGFBIp deposits in individuals with inherited corneal diseases caused by mutations in the *TGFBI* gene.

## Introduction

The transforming growth factor beta induced protein (TGFBIp; also known as kerato-epithelin, beta ig-h3, βig-h3, RGD-containing collagen-associated protein [RGD-CAP], and MP78/70) is an extracellular matrix protein encoded by the transforming growth factor beta induced gene (*TGFBI*; formerly designated *BIGH3*), which was first discovered in a lung adenocarcinoma cell line exposed to transforming growth factor beta [1]. The protein is composed of 683 residues including a 23-amino acid signal peptide. TGFBIp is highly conserved and the full-length human and porcine TGFBIp sequences are 93% identical [2]. TGFBIp contains an RGD motif (residues Arg_619_-Gly_620_-Asp_621_ in mature TGFBIp) and has been shown to interact with different integrins [3-7], fibronectin [8], collagen types I, II, IV, and VI [9,10] and to stimulate cell migration. Thus, the interaction of TGFBIp with these ligands suggests that TGFBIp plays a role in cell adhesion but its specific physiological role remains unclear.

The *TGFBI* gene is expressed in many tissues but TGFBIp is especially abundant in the cornea [11,12], skin [13], bone [14], cartilage [9], tendon [15], and kidney [16]. We discovered TGFBIp in the normal human cornea [12] and have recently purified and characterized the protein from human and porcine corneas [2]. Most of the corneal TGFBIp has a mature molecular mass of 68 kDa and migrates as an about 65 kDa protein in reducing sodium dodecyl sulfate-polyacrylamide gel electrophoresis (SDS-PAGE). The corneal TGFBIp is truncated in the COOH-terminus immediately after the putative integrin-binding sequence (RDG) and most likely lacks posttranslational modifications. In addition, about 60% of the corneal TGFBIp is covalently associated with insoluble components.

Mutations in the *TGFBI* gene are associated with an accumulation of TGFBIp in several inherited corneal disorders that lead to impaired vision [17-19]. Some mutations result in an accumulation of amyloid within the corneal stroma [18,20,21] while others generate fuschinophilic crystalloid deposits in the cornea that are characteristics of the granular corneal dystrophies. Furthermore, the Arg124His mutation gives rise to a combination of granular and amyloid deposits while mutation Arg555Gln causes curly fibers characteristic of Thiel-Behnke dystrophy [18]. In a recent systemic investigation of TGFBIp deposits in a patient with lattice corneal dystrophy type I, deposits were not detected in any tissue except the cornea, suggesting a cornea-specific mechanism for the TGFBIp accumulations [22].

The *TGFBI* gene is highly expressed in the corneal epithelium [11] and stromal cells [23], suggesting that wild-type and mutant TGFBIp are synthesized locally in normal and diseased corneas. However, significant amounts of TGFBIp have been reported in plasma from a healthy person and an individual with granular corneal dystrophy [24] using the only antiserum against TGFBIp available at the time (anti-p68^βig-h3^) [25]. This suggested that at least some corneal TGFBIp may originate from plasma. That some corneal proteins are derived from the plasma is well established. Thus, bioinformatic comparison of the cornea proteome [26] with high quality gene expression data from the cornea strongly indicates that most plasma proteins are not synthesized in the cornea [27] but probably enter this tissue from neighboring blood vessels. The possibility that TGFBIp deposition in the *TGFBI* corneal disorders might be plasma derived was supported by the somewhat comparable human disease hypergammaglobulinemia. Immunoglobulin normally enters the cornea from plasma and in hypergammaglobulinemia, this protein sometimes aggregates and deposits in the cornea [28-32].

However, recently we have noticed that the anti-p68^βig-h3^ utilized to immunodetect TGFBIp in plasma reacted with another protein, raising doubt on the conclusion that human plasma contains significant amounts of TGFBIp [24]. We hence examined the specificity of the previously utilized antiserum, anti-p68^βig-h3^. Here, we report that it is not only specific against TGFBIp but also reacts with human keratin-1, a common environmental protein contaminant having approximately the same molecular mass as TGFBIp. Using a new and highly specific antiserum (anti-TGFBIp^134-236^) for immunodetection of TGFBIp, we conclude that the previous strong detection of TGFBIp in normal human plasma was in error and that TGFBIp is not detectable in normal human plasma under the conditions tested. Thus, our results suggest that corneal TGFBIp is not imported into the cornea from plasma but originates from local synthesis in the cornea.

## Methods

### Cloning and purification of the NH_2_-terminal transforming growth factor beta induced protein fragment

A human NH_2_-terminal 11.4 kDa TGFBIp-fragment covering residues Gly_134_-Ile_236_ of the full-length precursor protein (Gly_111_-Ile_213_ of the mature protein) was cloned by PCR using forward primer 5'- CAC CGG GCC CGG CAG CTT CAC CAT CTT CG-3', reverse primer 5'-TCA GAT GAC CTT ATC GAT CGA TGA GGT G-3', and human full-length *TGFBI* cDNA as template. The resulting PCR product was inserted into the expression plasmid pET100/D-TOPO (Invitrogen, Taastrup, Denmark) thereby introducing an NH_2_-terminal His_6_-tag. *E. coli* strain TOP10F' (Invitrogen, Taastrup, Denmark) was transformed with the plasmid and the transformed cells were selected on Luria Broth (LB) medium/agar plates. The sequence of the insert was verified by DNA sequencing.

*E. coli* strain BL21 Star^TM^ (DE3) from Invitrogen (Taastrup, Denmark) was transformed with the plasmid and cultured in LB medium at 37 °C. Protein expression was induced by adding isopropyl-beta-D-thiogalactopyranoside (IPTG) to a final concentration of 1 mM and the cells were grown for two h. Cells were harvested by centrifugation, suspended in 40 ml Tris-buffered saline (TBS; 20 mM Tris-HCl, pH 7.6, and 137 mM NaCl) and lysed three times in a French press. The crude cell extract was centrifuged at 22,000x g for 30 min at 4 °C and the insoluble fraction was dissolved at 4 °C in 8 ml 20 mM Tris-HCl (pH 7.4), 10 mM imidazole, and 6 M guanidine, before being centrifuged at 16,000x g for 20 min. The resulting supernatant was filtered and loaded onto a HiTrap Ni^2+^-affinity column (Amersham Biosciences, Little Chalfont, England) and the column was washed thoroughly with 20 mM Tris-HCl (pH 7.4), 500 mM NaCl, 10 mM imidazole, and 6 M guanidine. The His-tagged NH_2_-terminal fragment of TGFBIp was eluted stepwise with 20, 40, 60, and 100 mM imidazole in the same buffer. Each fraction was dialyzed into 20 mM ammonium bicarbonate and precipitated proteins were analyzed by reduced SDS-PAGE. The 60 mM and 100 mM eluates were dried in a speed vac and resuspended in 50 mM Hepes (pH 7.4) and 100 mM NaCl.

### Production of antiserum (anti-TGFBIp^134-236^) and determination of detection limit

Rabbits were immunized five times subcutaneously with approximately 60 μg of the NH_2_-terminal TGFBIp-fragment per injection at four-week intervals. To determine the detection limit of the anti-TGFBIp^134-236^ antiserum, immunoblotting was performed with increasing amounts (2 ng-0.5 μg) of porcine corneal TGFBIp that had been purified as previously described [2].

### Depletion of albumin and IgG from human plasma

Normal human plasma obtained from the Danish National Serum Institute, Copenhagen was depleted of albumin and IgG by affinity chromatograpy. First, albumin was depleted using agarose immobilized Anti-HSA Affibody molecule (Affibody AB, Bromma, Sweden) [33,34] following the manufacturer's recommended protocol. Secondly, the flow-through was diluted five times in 20 mM NaH_2_PO_4_ (pH 7.0) and applied to a Protein G Sepharose^TM^ 4 Fast Flow resin (Amersham Biosciences, Little Chalfont, England) equilibrated in the same buffer. The flow-through was dialyzed into 10 mM ammonium bicarbonate, lyophilized in a speed vac, and dissolved in 20 mM Tris-HCl (pH 7.4) and 100 mM NaCl to a final volume equaling that of the crude plasma. The column materials used for the removal of albumin and IgG did not have affinity for TGFBIp (data not shown).

### SDS-polyacrylamide gel electrophoresis (SDS-PAGE)

Blank samples containing SDS sample buffer and increasing amounts of dithiothreitol (DTT; 0-50 mM) were prepared and boiled to investigate the cross-reactivity of the anti-p68^βig-h3^ antiserum directed against TGFBIp-fragment Val_210_-His_683_ of the precursor protein. The anti-p68^βig-h3^ antiserum was the first antiserum generated against TGFBIp [25]. The plasma samples and purified corneal TGFBIp were boiled in SDS sample buffer containing 10 mM DTT. All samples were analyzed on 5-15% linear gradient gels (10x10x0.1 cm) using the 2-amino-2-methyl-1,3 propandiol (ammediol)/glycine/HCl buffer system as described by Bury [35].

### Immunoblotting

Following SDS-PAGE, proteins were electroblotted to a polyvinylidene difluoride (PVDF) membrane (Millipore Immobilon transfer membranes, Millipore, Bedford, MA) and analyzed by immunoblotting. The PVDF membranes were blocked in 30 ml 1% dry milk solution in Tris-buffered saline with 0.1% Tween (TBS-T; 20 mM Tris-HCl, pH 7.6, 137 mM NaCl, and 0.1% Tween) for one h at room temperature before the rabbit antiserum against human TGFBIp was added to the blocking solution. The previous utilized anti-p68^βig-h3^ antiserum [24] was diluted 1:20,000, while the anti-TGFBIp^134-236^ antiserum against the NH_2_-terminal fragment of TGFBIp was diluted 1:2,000. After an overnight incubation with the primary antibody at 4 °C, the membrane was washed 3x15 min with TBS-T and then incubated for two h at room temperature in 60 ml of TBS-T containing 2 μl of anti-rabbit IgG peroxidase conjugate (Sigma Chemical Co., St. Louis, MO) and washed 3x15 min with TBS-T. Finally, the membranes were developed for one min using the enhanced chemiluminescence western blotting detection system and reagents (Amersham Biosciences, Little Chalfont, England). Purified TGFBIp from porcine corneas was used as a control.

### Collection and analysis of dust

Normal environmental dust collected from three locations in the laboratory was pooled, and suspended in sample buffer containing 35 mM DTT to a final and absolute concentration of 50 mg/ml. The suspension was boiled for five min and centrifuged, then the supernatant (stock) was analyzed by SDS-PAGE.

### Protein visualization and identification by matrix-assisted laser desorption ionization mass spectrometry

Proteins separated by SDS-PAGE were visualized by silver or coomassie brilliant blue staining as indicated. The protein bands of interest were excised and prepared for matrix-assisted laser desorption ionization mass spectrometry (MALDI) analysis as previously described for 2-D gel spots [36]. MALDI-time of flight mass spectrometry (MALDI-TOF MS) data was acquired using a Quadrupole-time of flight (Q-TOF) Ultima Global instrument (Micromass/Waters Corp., Manchester, United Kingdom) and the peak list of peptides were used to query all entries of the Swiss-Prot protein database on a local Mascot server using the Mascot search engine (Matrix Sciences, London, United Kingdom). The searches were performed with a peptide mass tolerance of 50 parts per million (ppm). Propionamide was selected as fixed modification of cysteine residues, and oxidation of methionine residues was selected as variable modification in the searches. In addition, a single missed tryptic cleavage was allowed. Only significant hits as defined by Mascot probability analysis were accepted.

## Results

**Figure 1:**
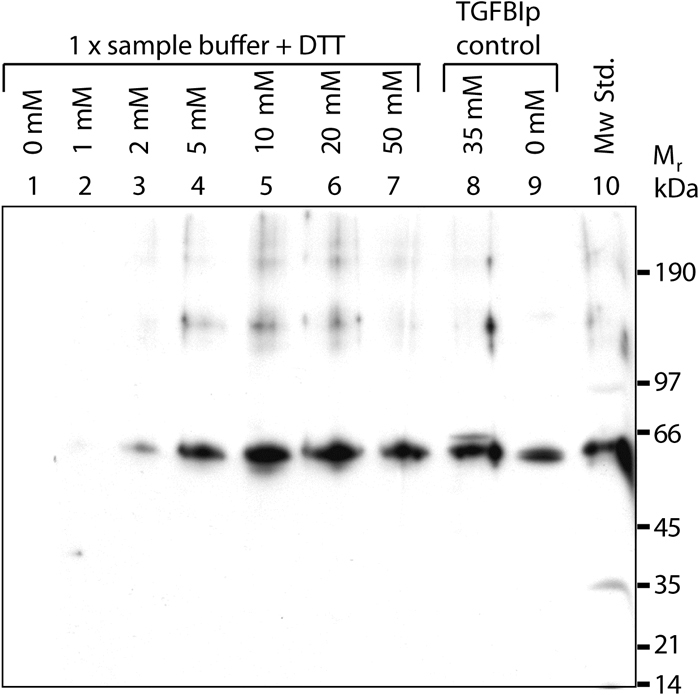
The anti-p68^βig-h3^ antiserum shows strong reactivity with a protein-contaminant under reducing conditions. The immunoblot shows sample buffer as blank samples containing increasing amounts of DTT. The anti-p68^βig-h3^ antiserum against residues Val_210_-His_683_ of the TGFBIp precursor was used in a dilution ratio of 1:20,000. Lanes 1-7 contain sample buffer with increasing amounts of DTT (0-50 mM). Lane 8 shows 0.16 μg of TGFBIp in sample buffer containing 35 mM DTT. Lane 9 shows 0.16 μg of TGFBIp in sample buffer in the absence of DTT. Lane 10 is the molecular weight standard.

Recent observations in our laboratory have indicated that the anti-p68^βig-h3^ antiserum generated against residues Val_210_-His_683_ of the precursor TGFBIp strongly reacts against another protein. Therefore, the specificity of this antiserum was analyzed by immunoblotting using sample buffer as blank samples with increasing concentrations of DTT (Figure 1, lanes 1-7). Using non-reducing conditions, the antiserum did not react with the blank sample (Figure 1, lane 1). However, the antiserum reacted strongly with a protein of about 65 kDa as the concentration of DTT was increased (Figure 1, lanes 2-7). In addition, the reactivity of the anti-p68^βig-h3^ antiserum against samples containing TGFBIp was tested under both reducing and non-reducing conditions (Figure 1, lanes 8 and 9). As expected, the antiserum showed strong reactivity against a protein contaminant (about 65 kDa) in the presence of 35 mM DTT (Figure 1, lane 8). Under non-reducing conditions (Figure 1, lane 9) only TGFBIp (about 64 kDa) is recognized by the anti-p68^βig-h3^ antiserum. Thus, this antiserum reacted strongly with a protein contaminant following reduction of disulfides, which also explains the apparent reaction with the molecular weight marker (Figure 1, lane 10).

**Figure 2:**
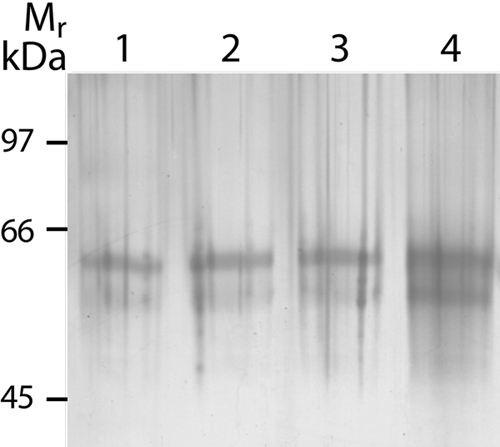
Analysis and identification of the environmental protein contaminants. Silver stained SDS gel of blank and dust samples. Lanes 1-3 show blank samples containing sample buffer and 35 mM DTT. Lane 4 shows proteins extracted from 25 μg of normal environmental dust in sample buffer containing 35 mM DTT. The upper band (about 65 kDa) is keratin-1 and the lower band (60 kDa) is keratin-10.

To determine the identity and origin of the approximately 65 kDa contaminating protein recognized by the antiserum, blank samples (Figure 2, lanes 1-3) and normal environmental dust that was dissolved in sample buffer containing 35 mM DTT (Figure 2, lane 4) were separated by SDS-PAGE and the proteins were visualized by silver staining. Both the blank and dust samples contain two predominant proteins with molecular weights of 60 kDa and about 65 kDa. However, the protein bands were more intense in the lane with dissolved dust than in the blank samples. The 60 kDa and 65 kDa proteins were identified by MALDI-MS as human keratin-10 (Protein score: 119) and keratin-1 (Protein score: 93), respectively. Keratin-1 has a theoretical molecular weight of 66.1 kDa, while keratin-10 has a theoretical molecular weight of 59.8 kDa.

**Figure 3:**
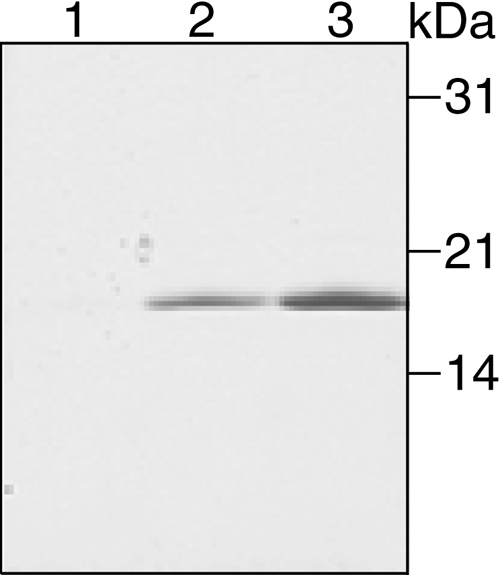
Purification of recombinant NH_2_-terminal TGFBIp fragment. The His-tagged NH_2_-terminal TGFBIp-fragment (residues Gly_134_-Ile_236_) was expressed in *E. coli* and purified from the insoluble fraction using Ni^2+^-affinity chromatography. The step-wise elution was analyzed by SDS-PAGE and the gel was stained with coomassie brilliant blue. Lane 1 shows the eluate from elution buffer containing 40 mM imidazole. Lane 2 contains the eluate from 60 mM imidazole. Lane 3 contains the eluate from 100 mM imidazole. All lanes contain 50 μl of the elution.

Due to this observation, a new antiserum against human TGFBIp (anti-TGFBIp^134-236^) was produced to detect and determine the concentration of TGFBIp in plasma. An NH_2_-terminal fragment constituting residues Gly_111_-Ile_213_ of the mature protein (equaling Gly_134_-Ile_236_ of the full-length protein) was cloned and expressed in *E. coli*. The purification from the insoluble fraction of the induced *E. coli* cells was performed under denaturing conditions. The Ni^2+^-affinity chromatography resulted in a highly pure elution of the His-tagged fragment (Figure 3), which was used to immunize rabbits.

**Figure 4:**
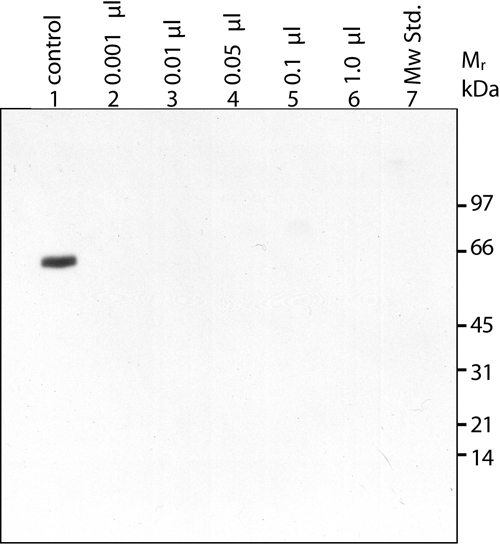
Immunoblotting of crude human plasma using the specific anti-TGFBIp^134-236^ antiserum. The immunoblot shows increasing amounts of crude human plasma diluted in 20 mM Tris-HCl (pH 7.4) and 100 mM NaCl. Each lane contains 20 μl of diluted crude plasma. Lane 1 contains 0.2 μg of purified porcine TGFBIp was used as a control. Lanes 2-6 have increasing amounts of crude human plasma. The indicated volumes refer to the amount of crude plasma. Lane 7 is the molecular weight standard.

To detect TGFBIp in human plasma, increasing amounts of normal crude plasma (1 nl-1 μl) were analyzed by immunoblotting following reducing SDS-PAGE. Purified TGFBIp from porcine corneas was used as a control (Figure 4). The anti-TGFBIp^134-236^ antiserum did not react with any proteins in crude plasma but did react with purified porcine TGFBIp. Thus, as expected from the high sequence identify (98%) between the NH_2_-terminal fragments of human and porcine TGFBIp [2], the TGFBIp antiserum recognized porcine TGFBIp.

**Figure 5:**
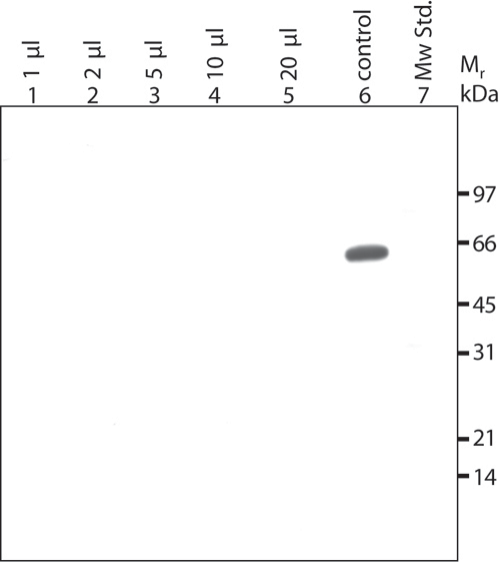
Immunoblotting of albumin/IgG-depleted human plasma using the specific anti-TGFBIp^134-236^ antiserum. The immunoblot shows increasing amounts of albumin/IgG-depleted human plasma in 20 mM Tris-HCl (pH 7.4) and 100 mM NaCl. Each lane contains 20 μl of diluted albumin/IgG-depleted plasma. Lanes 1-5 contain increasing amounts of albumin/IgG-depleted human plasma. The indicated volumes refer to the equaling amount of crude plasma. Lane 6 contains 0.2 μg of purified porcine TGFBIp, which was used as a control. Lane 7 is the molecular weight standard.

Albumin (67 kDa) and IgG comprise 55% [37] and 10-25% [38] of the total plasma protein mass, respectively. Thus, depletion of albumin and IgG from plasma should remove 65-80% of the total protein content allowing larger volumes of plasma to be analyzed by SDS-PAGE. Consequently, this will enhance the detection of TGFBIp if present in plasma. The immunoblotting was repeated using increasing amounts of the albumin/IgG-depleted plasma preparation equaling 1-20 μl of crude plasma. Despite the high amounts of plasma loaded on the gel, no reaction was observed (Figure 5).

**Figure 6:**
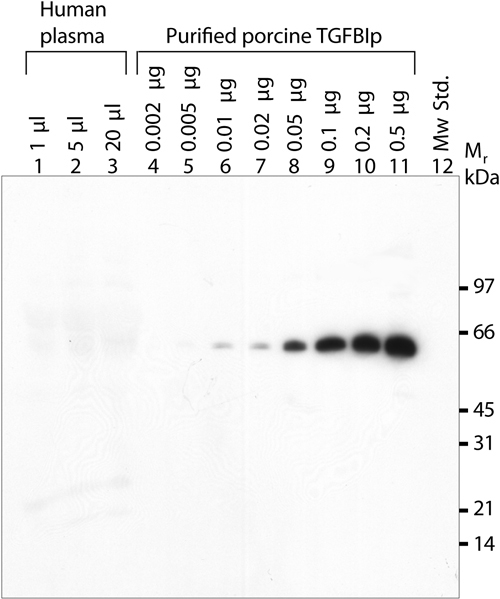
Detection limit of TGFBIp using purified porcine TGFBIp from corneas. The immunoblot shows increasing amounts of albumin/IgG-depleted human plasma and purified porcine TGFBIp (2-500 ng). Lanes 1-3 show increasing amounts of albumin/IgG-depleted human plasma. The indicated volumes refer to the equaling amount of crude plasma. Lanes 4-11 show increasing amounts of purified porcine TGFBIp. The amount of loaded porcine TGFBIp is indicated for each lane. The detection limit is about 10 ng TGFBIp. Lane 12 is the molecular weight standard.

The antiserum against the NH_2_-terminal fragment of human TGFBIp shows high specificity and its sensitivity was determined using increasing amounts of purified porcine corneal TGFBIp (Figure 6). In addition, increasing amounts of the albumin/IgG-depleted human plasma was analyzed on the same gel to verify the results depicted in Figure 5. The detection limit of TGFBIp using the present immunoblotting method was about 10 ng. Since no TGFBIp was detected in an albumin/IgG-depleted sample equaling 20 μl of crude plasma, the present results show that the concentration of TGFBIp in plasma is less than 0.5 mg/l. This estimation is based on the assumption that the antiserum reacts equally with human and porcine TGFBIp, which seems reasonable owing to the 98% identity of the NH_2_-terminal fragments of TGFBIp.

## Discussion

In an earlier study using immunoblotting and a polyclonal antibody against a recombinant fragment of TGFBIp (Val_210_-His_683_), our laboratory reported the presence of significant amounts of TGFBIp in human plasma from both normal individuals and a patient suffering from granular corneal dystrophy [24]. However, the specificity of this antiserum, anti-p68^βig-h3^, is now questioned as it reacts not only against TGFBIp but also with an environmental protein contaminant that is present even in blank samples only containing SDS sample buffer and DTT. In this study, we have shown that the anti-p68^βig-h3^ antiserum is not specific for TGFBIp because it reacts strongly with human keratin-1 under reducing conditions. Unfortunately, keratin-1 migrates at the same position (about 65 kDa) as TGFBIp under reducing conditions indicating that the previous interpretation of finding a significant amount of TGFBIp in human plasma was in error [24]. Human keratins such as keratin-10 (59.8 kDa), keratin-9 (62.1 kDa), epidermal keratin-2 (65.9 kDa), and keratin-1 (66.1 kDa) are known to be common laboratory contaminants and an analysis by use of normal dust verified that human keratin-1 and -10 are indeed abundant in dust. Therefore, the trace amount of the keratin-10/1 pair in blank control samples probably originated from dust. Keratins are intermediate filament proteins forming approximately 10 nm cytoskeletal filaments that stabilize the structure of the cell. The keratin-10/1 pair is tissue-specific and mainly expressed in suprabasal differentiating keratinocytes within epidermis. Thus, keratin-1 and keratin-10 together with epidermal keratin-2 are characteristic of the post-mitotic upper layer of epidermis and therefore likely to end up in dust as the cornified and dead keratinocytes desquamate from the skin [39]. Since keratin-1 and the recombinant TGFBIp-fragment (Val_210_-His_683_ of TGFBIp) show no significant sequence similarity, it is very unlikely that antibodies will recognize both proteins through reactivity with shared epitopes. However, contamination of an antigen preparation with small amounts of dust prior to immunizations could lead to reactivity of the antiserum against keratin-1 in addition to TGFBIp. Since both mature TGFBIp [2] and keratin-1 migrate with molecular masses of about 65 kDa in reducing SDS-PAGE, reactivity against contaminating keratin-1 in immunoblotting is easily misinterpreted as detection of TGFBIp. The fact that we observe reactivity of the anti-p68^βig-h3^ antiserum against keratin-1 only under reducing conditions is probably because all keratins of the cornified layer of epidermis are cross-linked by intermolecular disulfide bonds forming high molecular weight structures [40], which prevent the protein from entering the gel under non-reducing conditions. Thus, we conclude that the previous strong detection of TGFBIp in plasma by immunoblotting [24] was incorrect, demonstrating the importance of using direct protein identification methods such as mass spectrometry or NH_2_-terminal sequencing. Because of this we have produced a new antiserum against TGFBIp and reinvestigated the presence of TGFBIp in normal human plasma with the aim of determining its concentration. With this anti-TGFBIp^134-236^ antiserum, which showed no indications of reactivity with other proteins, we could not detect TGFBIp in normal crude plasma or in plasma depleted of albumin and IgG under the conditions tested. Based on the detection limit, which was estimated using purified TGFBIp from porcine corneas, we conclude that the concentration of TGFBIp in plasma is below 0.5 mg/l, which is in the range of so-called tissue leakage proteins caused, for example, by tissue turnover and thus with no physiological function in plasma [37]. Thus, our observation is consistent with another study reporting the detection of TGFBIp in the μg/ml range in serum from healthy individuals and patients with colorectal cancer using an ELISA assay [41].

The present evidence against a plasma derived origin for corneal TGFBIp has implications for the pathogenesis of inherited corneal disorders caused by mutations in the *TGFBI* gene. It suggests that corneal TGFBIp deposits in individuals with mutations in the *TGFBI* gene originate from local synthesis in the cornea rather than from the plasma as in hypergammaglobulinemia.
